# Physician experience and rates of plasma HIV-1 RNA suppression among illicit drug users: an observational study

**DOI:** 10.1186/1471-2334-12-22

**Published:** 2012-01-25

**Authors:** Sassan Sangsari, M-J Milloy, Amir Ibrahim, Thomas Kerr, Ruth Zhang, Julio Montaner, Evan Wood

**Affiliations:** 1 Department of Integrated Sciences, University of British Columbia, Vancouver, BC, Canada; 2 British Columbia Centre for Excellence in HIV/AIDS, St. Paul's Hospital, 608-1081 Burrard Street, Vancouver, BC, Canada V6Z 1Y6; 3 School of population and public health, University of British Columbia, Vancouver, British Columbia, Canada; 4 Department of Medicine, University of British Columbia, Vancouver, British Columbia, Canada

## Abstract

**Background:**

Despite the availability of antiretroviral therapy (ART), suboptimal treatment outcomes have been observed among HIV-seropositive illicit drug users. As there is an urgent need to improve responses to antiretroviral therapy among this population, we undertook this study to evaluate the role of physician experience on rates of plasma HIV-1 RNA suppression following initiation of ART.

**Methods:**

Using data from a community-recruited cohort of HIV-positive illicit drug users, we used Cox proportional hazards regression to model the time to plasma viral HIV RNA < 500 copies/mL among antiretroviral-naïve subjects initiating ART. Physician experience was defined as a continuous variable measured per 100 HIV-infected patients previously enrolled in the province-wide HIV treatment registry by that physician at the time a patient was enrolled.

**Results:**

Between May 1996 and December 2008, 267 individuals initiated ART among whom 227 (85%) achieved a plasma HIV RNA < 500 copies/mL during the study period. In a multivariate analysis, greater physician experience was independently associated with higher rates of plasma HIV RNA suppression (adjusted hazard ratio [AHR] = 1.17, 95% confidence interval [CI]: 1.03-1.34) after adjustment for adherence to ART. Other factors associated with viral suppression included engagement in methadone maintenance therapy (AHR = 1.61, 95% CI: 1.23-2.09), ≥ 95% adherence to ART (AHR = 2.42, 95% CI: 1.80-3.26), baseline CD4 count (AHR = 0.89, 95% CI: 0.83-0.96) and baseline plasma HIV-1 RNA (AHR = 0.65, 95% CI: 0.53-0.81).

**Conclusions:**

In this setting of universal HIV/AIDS care, illicit drug users with more experienced physicians exhibited faster rates of plasma viral load suppression. These findings argue for specialized services to help optimize HIV treatment outcomes among this population.

## Background

With over 30 million cases distributed worldwide, the HIV/AIDS pandemic is a global public health emergency [1]. In areas outside sub-Saharan Africa, nearly one in three new infections occur among individuals who use illicit drugs [2]. Current treatment guidelines recommend the initiation of antiretroviral therapy in order to durably suppress HIV-1 plasma HIV RNA levels in order to reduce morbidity, mortality and the risk of HIV transmission [3].

Despite the availability of effective treatment, several individual, social and structural factors have posed barriers to effective ART outcomes among illicit drug users. While access and adherence to the prescribed drug regimen is recognized as the most important determinant of treatment success [4], other factors, including clinical status at treatment initiation, specific illicit drug use patterns [5] and homelessness [6], may also play a role. Less well evaluated is the role of healthcare factors, such as the role of prescribing physicians. Previous studies have indicated that physicians may be less willing to prescribe ART to illicit drug users [7]. However, the effect of provider characteristics on outcomes from ART has not been previously examined among drug-using HIV-infected populations. Therefore, we examined the influence of physician experience on achieving plasma viral HIV-1 RNA < 500 copies/mL among injection drug users initiating ART.

## Methods

Data for this study were obtained from the AIDS Care Cohort to evaluate Exposure to Survival Services (ACCESS), an ongoing observational prospective cohort of HIV-positive illicit drug users [8,9]. Following recruitment through street outreach and the provision of informed consent, participants provide a blood sample and complete an extensive interviewer-administered questionnaire as well as a nurse-administered examination. Follow-ups occur semi-annually. Most of these individuals reside in the Downtown Eastside (DTES) neighbourhood, an area in Vancouver, Canada, with high prevalence of illicit drug use, poverty and homelessness, as well as HIV and hepatitis C infection [8,9]. Individuals from the ACCESS cohort were aged 18 years or older at recruitment, tested seropositive to HIV-1 and had used non-cannabinoid illicit drugs in the month prior to enrolment. ACCESS has been approved by the University of British Columbia/Providence Healthcare Research Ethics Board.

Data on HIV clinical monitoring and drug-using behaviour were augmented with information on HIV care and treatment outcomes from the province-wide centralized ART dispensary and HIV clinical monitoring laboratory at the British Columbia Centre for Excellence in HIV/AIDS (BC-CfE) [8,9]. As a result, we had access to a complete profile of CD4 cell count and plasma HIV-1 RNA level for each participant. ART adherence was defined as the number of days ART was dispensed over the number of days an individual was eligible for ART in the previous 6 months; the resulting proportion was dichotomized as > 95% vs. ≤ 95% adherence. Previous studies have validated this method of measuring ART adherence using pharmacy refill data [10]. Measurements of plasma HIV-1 RNA were obtained using the Roche Amplicor Monitor assay (Roche Molecular Systems, Mississauga, Canada.)

This study included all ACCESS participants who were ART-naïve at recruitment, initiated treatment during the study period, and had at least one clinical test measuring CD4 cell count and plasma HIV-1 RNA level during the first 12 months on ART. Our main outcome, time to plasma HIV-1 RNA suppression, was operationalized as the date of the first observation with a plasma viral HIV-1 RNA < 500 copies/mL.

The primary explanatory variable of interest was physician experience, defined as the number of patients that the participant's prescribing physician had previously enrolled in the province-wide HIV treatment registry. This was considered as a continuous variable and, since physicians could become more experienced over time, was fixed for each participant at the time they initiated ART. In British Columbia, antiretroviral prescribing physicians could be located anywhere in the province and not at only one institution. Patients selected antiretroviral-prescribing primary care or specialist physicians in a non-random manner through self-selection or referral from other physicians. Physician experience was fixed as a baseline characteristic at the time the patient initiated ART as we believe that this was the best way to estimate physician experience. We recognized certain behaviours (e.g. drug use activity) could confound this association so these measures were treated as time-updated variables based on each participant's semi-annual follow up visit.

Several variables that could influence the association between physician experience and plasma HIV-1 RNA suppression were also assessed, including age, gender (female vs. male), Aboriginal ancestry (yes vs. no) and Downtown Eastside residence (yes vs. no). Clinical variables included ART adherence (≥ 95% vs. < 95% in first year of treatment), current enrolment in methadone maintenance therapy (no vs. yes), protease inhibitor as part of the first ART regimen (yes vs. no), CD4 cell count at baseline (per 100 cells), plasma HIV-1 RNA level at baseline (per log_10 _increase) and the year of ART initiation (per more recent year). Illicit drug use measures included daily cocaine use (yes vs. no) and daily heroin use (yes vs. no) and referred to the six-month period prior to the interview. Patients were prescribed antiretroviral therapy consistent with therapeutic guidelines which, beginning in 1996 for all patients, recommended triple combination therapy. While the drugs used over the study period changed markedly over time, consistent with previous analyses, we adjusted for whether protease inhibitors were used in the initial regimen or not as a strategy to adjust for confounding that could occur as a result of regimen type. Given the large number of nucleosides used in the backbone of the ART regimen during the study period, we elected to not adjust for this in the analysis.

We initially examined baseline characteristics of our cohort, including physician experience, and tested for significant differences using Pearson's *χ*^2 ^statistic. We subsequently used univariate and multivariate Cox proportional hazards regression to evaluate the impact of the variables considered on the time to plasma HIV-1 RNA < 500 copies/mL. The multivariate model was built using an *a priori *model building strategy developed by Greenland and colleagues [11]. This strategy aims to produce a parsimonious set of covariates to better estimate the adjusted relationship between a primary explanatory variable and an outcome of interest. It has previously been used, for example, to assess the independent relationship between incarceration patterns and non-adherence to ART among injection drug users [12]. To start, we fitted a multivariate model that included physician experience and the full set of secondary explanatory variables. After noting the value of the regression coefficient associated with physician experience in the full model, we used a manual stepwise approach to fit a series of reduced models, each with one secondary explanatory variable dropped from the full set. Comparing the value of the coefficient in the full model to the value of the coefficient for physician experience in each of the reduced models, we dropped the variable associated with the smallest relative change from further consideration. This iterative process was continued until the maximum change exceeded 5%. This technique has been employed in several studies to estimate the independent effect of an explanatory variable on an outcome of interest [12].

## Results

Between May 1996 and December 2008, 267 ART-naïve IDU who initiated ART over follow-up were recruited. This group included 124 (46.4%) women and 105 (39.3%) individuals who reported being of Aboriginal ancestry. The baseline characteristics of the study sample along with the median physician experience of different groups are presented in Table 1. As shown here, individuals engaged in methadone maintenance therapy had the most experienced providers (median = 60, interquartile range [IQR]: 20-144), while individuals residing outside of the Downtown Eastside were treated by providers with the least experience (median = 28, IQR: 8-92).

**Table 1:** Baseline characteristics of 267 HIV-seropositive active illicit drug users including physician experience

Characteristic	All (267)	Physician experience	*p*-value
		
	n (%)	Median (IQR^3^)	
Gender			

Male	143 (53.6)	41 (13-98)	0.434
	
Female	124 (46.4)	48 (13-130)	

Aboriginal ancestry			

No	162 (60.7)	55 (15-103)	0.716
	
Yes	105 (39.3)	42 (10-116)	

DTES residence			

No	83 (31.1)	28 (8-92)	0.018
	
Yes	184 (68.9)	56 (16-123)	

ART adherence			

≤ 95% in first year	186 (69.7)	42 (10-103)	0.078
	
> 95% in first year	81 (30.3)	58 (22-127)	

Methadone maintenance			

No	160 (59.9)	38 (11-90)	0.003
	
Yes	107 (40.1)	60 (20-144)	

Daily cocaine use^1^			

No	175 (65.5)	59 (13-126)	0.045
	
Yes	92 (34.5)	34 (12-92)	

Daily heroin use^1^			

No	196 (73.4)	47 (13-104)	0.658
	
Yes	71 (26.6)	43 (14-116)	

PI^2 ^in first ART regimen			

No	155 (58.1)	44 (14-105)	0.975
	
Yes	112 (41.9)	58 (12-111)	

1. Refers to the six-month period prior to baseline

2. Protease inhibitor

3. Inter-quartile range. Physician experience was defined as per 100 HIV-infected patients previously treated by that physician at the time each participant initiated ART.

During the study period, 227 participants (85.0%) achieved at least one observation of plasma HIV-1 RNA < 500 copies/mL, reflecting an incidence density of 65.2 per 100 person years (95% CI: 57.0-74.2). Table 2 presents the unadjusted hazard ratios (HR) of time to plasma HIV-1 RNA suppression by physician experience as well as other explanatory covariates. As shown, physician experience (per 100 patients previously enrolled) was significantly associated with time to suppression (HR = 1.25, 95% CI: 1.10-1.42, *p*-value < 0.001), as were older patient age (HR = 1.26 per 10 years, 95% CI: 1.08-1.47, *p*-value = 0.004), engagement in methadone maintenance therapy (HR = 1.54, 95% CI: 1.18-2.00, *p*-value = 0.001), ≥ 95% ART adherence (HR = 4.12, 95% CI: 3.10-5.54, *p*-value < 0.001), CD4 cell count (HR = 0.90, 95% CI: 0.84-0.96, *p-*value = 0.003), baseline plasma viral load (HR = 0.71, 95% CI: 0.58-0.87, *p*-value = 0.001), and the year of ART initiation (HR = 1.17 per more recent year, 95% CI: 1.12-1.21, *p*-value < 0.001.)

**Table 2:** Univariate and multivariate analyses of factors associated with time to HIV-1 RNA < 500 copies/mL among individuals beginning antiretroviral therapy

Characteristic	Hazard Ratio	**95% CI**^**3**^	*p*-value	Adjusted Hazard Ratio	**95% CI**^**3**^	*p*-value
Physician experience^1^						

Per 100 patients	1.25	1.10-1.42	< 0.001	1.17	1.04-1.34	0.031

Age^1^						

Per 10 years	1.26	1.08-1.47	0.004			

Gender^1^						

Female vs. male	0.95	0.73-1.24	0.711			

Aboriginal ancestry^1^						

Yes vs. no	1.05	0.80-1.36	0.103			

DTES residence^2^						

Yes vs. no	1.13	0.86-1.49	0.788			

Methadone maintenance^2^						

Yes vs. no	1.54	1.18-2.00	0.001	1.61	1.23-2.09	< 0.001

Daily cocaine use^2^						

Yes vs. no	0.92	0.68-1.24	0.578			

Daily heroin use^2^						

Yes vs. no	0.78	0.57-1.08	0.137			

ART adherence^2^						

> 95% vs. ≤ 95%	4.12	3.10-5.54	< 0.001	2.42	1.80-3.26	< 0.001

PI in first regimen^1^						

Yes vs. no	1.23	0.95-1.61	0.117			

CD4 cell count^1^						

Per 100 cells	0.90	0.84-0.96	0.003	0.89	0.83-0.96	0.001

Plasma viral load^1^						

Per log_10 _increase	0.71	0.58-0.87	0.001	0.65	0.53-0.81	< 0.001

Year of ART initiation^1^						

Per more recent year	1.17	1.12-1.21	< 0.001			

1. Time invariant, observed at baseline

2. Time varying, observed every 6 months

3. 95% Confidence Interval

Table 2 presents adjusted hazard ratios (AHR) of time to plasma HIV-1 RNA suppression by physician experience as well as other covariates. As shown here, physician experience was independently associated with plasma HIV-1 RNA suppression (AHR = 1.17, 95% CI: 1.03-1.34, *p*-value = 0.031). Other factors that were associated with time to plasma HIV-1 RNA suppression in this multivariate analysis included methadone maintenance therapy (AHR = 1.61, 95% CI: 1.23-2.09, *p*-value < 0.001), baseline CD4 count (AHR = 0.89, 95% CI: 0.83-0.96, *p*-value < 0.001), baseline log_10 _HIV-1 RNA (AHR = 0.65, 95% CI: 0.53-0.81, *p*-value < 0.001) and adherence to ART (AHR = 2.42, 95% CI: 1.80-3.26, *p*-value < 0.001). When we fit a multivariate model including the three main associations shown in Table 1, we found that physician experience was associated with higher rates of plasma HIV-1 RNA viral load suppression (AHR = 1.23 per 100 patients enrolled, 95% CI: 1.07-1.40, *p*-value = 0.003) after adjustment for Downtown Eastside residence, methadone maintenance therapy and daily cocaine injection.

In a sub-analysis, we explored the impact of physician experience on time to viral load suppression when physician experience was defined as a categorical variable using tertiles. In the first tertile, physicians had treated less than 22 patients previously and the median time to plasma HIV-1 RNA < 500 copies/mL was 9.5 months (95% CI: 4.4-14.5). In the middle tertile, physicians had treated 22-81 patients previously and the median time to suppression was 6.2 months (95% CI: 3.0-13.4). In the most experienced tertile, physicians had treated greater than 81 patients previously and the median time to plasma HIV RNA suppression < 500 copies/mL was 3.0 months (95% CI: 2.3-4.7).

## Discussion

In this study, we found that greater physician experience was positively associated with the time to viral load suppression among drug users initiating ART. This association persisted in a multivariate model after adjusting for CD4 cell count, baseline plasma HIV-1 RNA level, methadone use, and adherence following ART initiation.

Our finding that physician experience was associated with higher rates of plasma HIV-1 RNA suppression is noteworthy. Prior to the advent of combination antiretroviral therapy, a US study found that higher physician experience was associated with longer patient survival [13]. Similar results have also been reported in the era of combination antiretroviral therapy [14]. Previous studies have also shown that negative attitudes among physicians towards HIV-positive drug users are linked to lower quality of care [7]. At the same time, the more drug users a physician has seen, the more likely he or she is to report a positive attitude toward injection drug users [7]. Previous studies also suggest that physicians often base their judgment regarding patient's level of adherence on non-medical clues such as socio-demographic criteria [15]. At the same time, attempts of clinicians to predict patient adherence to ART pre-initiation are often incorrect [16]. Less experienced physicians may carry a preconceived belief that drug users tend to be non-adherent. Such usage of extra-medical cues, even if shown to be indicative at times [17], could potentially lead to a patient's mistrust in the health care provider and facilitate a decline in the quality of doctor-patient communication [18].

Although our measure of adherence has been previously validated to predict plasma HIV RNA responses [10], CD4 count responses [19] and patient survival [9], it is possible that residual confounding related to patient adherence--and possibly mediated by physician experience--explain our findings. Specifically, it is possible that having an experienced physician contributed to positive daily adherence behaviour that was not captured by pharmacy refill. In addition, while this study demonstrated an association between physician experience and higher rates of plasma HIV RNA suppression, we were not able to explore explanations for this association. It is likely that physicians with greater HIV-related experience were able to more skilfully address drug toxicities and other concerns that may limit patient adherence.

Our study has some limitations to be noted. First, our cohort was not randomly selected and therefore may not be representative of all HIV-infected drug users in Vancouver or elsewhere. Second, although physician experience was defined based on an HIV treatment registry, for other variables we relied on measures of self-report, and therefore response bias could have undermined our ascertainment of sensitive behaviours, including illicit drug use and addiction treatment use. Finally, our analyses only includes follow-up information until 2008 and data collected when the lower limit of detection was 500 copies/mL. Future studies should continue to assess whether physician experience is associated with outcomes from HIV treatment, especially as modern HAART regimens are increasingly simple to take.

## Conclusions

In summary, we examined plasma HIV RNA responses after the initiation of ART among a long-running community-recruited prospective cohort of HIV-positive illicit drug users and observed that lower HIV-related experience of physicians was independently associated with lower rates of plasma HIV RNA suppression. These findings argue for more specialized treatment approaches to address the HIV treatment needs of this challenging population.

## Competing interests

Julio Montaner is supported by the Ministry of Health Services and the Ministry of Healthy Living and Sport, from the Province of British Columbia; through a Knowledge Translation Award from the Canadian Institutes of Health Research (CIHR); and through an Avant-Garde Award (No. 1DP1DA026182-01) from the National Institute on Drug Abuse at the US National Institutes of Health. He has also received funding from Merck, Gilead and ViiV to support research into Treatment as Prevention.

## Authors' contributions

SS, M-JM, TK, EW and JM conducted the study. EW, TK, AI and SS conceived the analysis. RZ performed all statistical calculations. M-JM, AI and SS drafted the manuscript and integrated revisions from all other co-authors. All authors reviewed and approved the final draft.

## Pre-publication history

The pre-publication history for this paper can be accessed here:

http://www.biomedcentral.com/1471-2334/12/22/prepub
